# The Efficacy of Pulmonary Rehabilitation in Patients with Idiopathic Pulmonary Fibrosis

**DOI:** 10.3390/life13020403

**Published:** 2023-02-01

**Authors:** Hee Eun Choi, Tae Hoon Kim, Ji Hoon Jang, Hang-Jea Jang, Jisook Yi, So Young Jung, Dae-Wook Kim, Jae Ha Lee

**Affiliations:** 1 Department of Physical Medicine and Rehabilitation, Haeundae Paik Hospital, Inje University College of Medicine, Busan 48108, Republic of Korea; 2 Division of Pulmonology and Critical Care Medicine, Department of Internal Medicine, Haeundae Paik Hospital, Inje University College of Medicine, Busan 48108, Republic of Korea; 3 Department of Radiology, Haeundae Paik Hospital, Inje University College of Medicine, Busan 48108, Republic of Korea; 4 Division of Dermatology, Haeundae Paik Hospital, Inje University College of Medicine, Busan 48108, Republic of Korea; 5 Department of Orthopedic Surgery, Haeundae Paik Hospital, Inje University College of Medicine, Busan 48108, Republic of Korea

**Keywords:** idiopathic pulmonary fibrosis, pulmonary rehabilitation, clinical efficacy, functional performance, exercise-related oxygen saturation, health-related quality of life

## Abstract

**Background:** This study evaluated the efficacy and safety of pulmonary rehabilitation (PR) on functional performance, exercise-related oxygen saturation, and health-related quality of life among patients with idiopathic pulmonary fibrosis (IPF). **Methods:** A total of 25 patients with IPF (13 in the PR group and 12 in the non-PR group) were enrolled between August 2019 and October 2021 at Haeundae-Paik Hospital in the Republic of Korea. A cardiopulmonary exercise test (CPET), six-minute walk test (6MWT), pulmonary function test (PFT), Saint George’s Respiratory Questionnaire (SGRQ), muscle strength test, and bioelectrical impedance analysis were performed in each group at baseline and after eight weeks of PR. **Results:** The mean age was 68 years of age and most subjects were male. Baseline characteristics were similar between the two groups. The distance during 6MWT after PR was significantly improved in the PR group (inter-group *p*-value = 0.002). VO_2_max and VE/VCO_2_ slopes showed a significant difference after eight weeks only in the PR group, but the rate of change did not differ significantly from the non-PR group. Total skeletal muscle mass, PFT variables, and SGRQ scores did not differ significantly between the groups. **Conclusions:** PR improved exercise capacity, as measured using CPET and 6 MWT. Further studies in larger samples are needed to evaluate the long-term efficacy of PR in IPF patients.

## 1. Introduction

Idiopathic pulmonary fibrosis (IPF) is a specific form of interstitial lung disease (ILD) characterized by a chronic, progressive, and variable clinical course. It occurs most often in elderly adults and is defined by histopathologic and/or radiologic features of usual interstitial pneumonia [1]. IPF has a poor prognosis, with a median survival of approximately 3 years, and is associated with morbidity [2]. 

IPF also causes significant problems in daily life, including limited movement, chronic and progressive dyspnea during exercise, fatigue, decreased exercise capacity, and decreased quality of life [3]. Although IPF is chronically progressive, antifibrotic therapy halves the decline in lung function and is effective in preventing acute exacerbation [4,5]. However, the effect of antifibrotic therapy on symptom improvement and exercise capacity is limited, and evidence for other non-pharmacologic treatments is lacking [6]. 

Along with anti-fibrotic drugs, international guidelines recommend vaccination against coronavirus disease 2019, pneumococcus, and seasonal influenza, supplemental oxygen, and pulmonary rehabilitation (PR) for patients with IPF [7,8]. PR is the most commonly administered and representative non-pharmacological treatment as “an evidence-based, multidisciplinary, and comprehensive intervention for patients with chronic respiratory disease who are symptomatic and often have reduced capacity for the activities of daily living” [9]. Among chronic obstructive pulmonary disease (COPD) patient cohorts, PR programs have been shown to be effective in improving exercise capacity, dyspnea, and health-related quality of life as drug-based therapeutic options [10]. However, compared with other chronic respiratory diseases, studies demonstrating the effectiveness of PR for IPF patients are still inadequate [6]. We hypothesized that PR would be useful for improving the functional capacity and quality of life of patients with IPF. In this study, we evaluated the efficacy and safety of PR for patients with IPF in terms of functional performance, exercise-related oxygen saturation, and health-related quality of life. In addition, we evaluated the safety of our PR program, which consists of individual exercise prescriptions using cardiopulmonary exercise tests.

## 2. Materials and Methods

### 2.1. Subjects

This was a prospective, interventional, single-center study of patients with IPF. From August 2019 to October 2021, patients with IPF that visited the Department of Pulmonology of Haeundae Paik Hospital in South Korea were enrolled. The non-PR group contained patients who fulfilled the inclusion criteria and agreed to participate in this study but could not perform the PR program. Patients in the non-PR group were recruited and matched with the PR group for age (range: ±5 years) and sex. The inclusion criteria were as follows: (1) patients diagnosed with IPF through multidisciplinary discussion and concordance with the clinical and radiological features in the guidelines of the American Thoracic Society/European Respiratory Society/Japanese Respiratory Society/Latin American Thoracic Association (ATS/ERS/JRS/ALAT) [1]; (2) patients within two years of their initial diagnosis of IPF; (3) patients who could walk on a treadmill; (4) patients who could cooperate with PR treatment; and (5) adults between the ages of 19 and 80 years who voluntarily consented to the purpose and method of this study. The exclusion criteria were (1) patients with a history of cerebrovascular disease or accompanying disease that limited exercise training; (2) patients with a high risk of cardiovascular accidents during exercise training [10]; (3) patients with neurological diseases or orthopedic diseases that prevented exercise training from being performed; (4) patients with an acute exacerbation of IPF; (5) patients who previously participated in a PR program; (6) patients with exercise stress testing contraindications; and (7) patients who refused to provide informed consent for the study. 

The PR group completed a comprehensive PR program for eight weeks, along with evaluation before and after the PR. The non-PR group received one exercise training session and was recommended to exercise and performed the same evaluation before and after an eight-week period.

This study was approved by the Institutional Review Board of Haeundae-Paik Hospital (approval no. 2019-10-022). All subjects were informed of the study protocol and provided written informed consent for their participation. 

### 2.2. Exercise Training

Each session of the PR program (participants completed 3 sessions per week) consisted of breathing retraining and chest expansion exercise for 10 min, aerobic exercise for 47 min, and resistance exercise for 10 min, in that order.

Breathing retraining included diaphragmatic breathing, segmental breathing, cough training, and inspiratory/expiratory muscle strengthening training using a Threshold IMT^®^/PEP^®^ (Philips Respironics, Murrysville, PA, USA) instrument.

**Figure 1:**
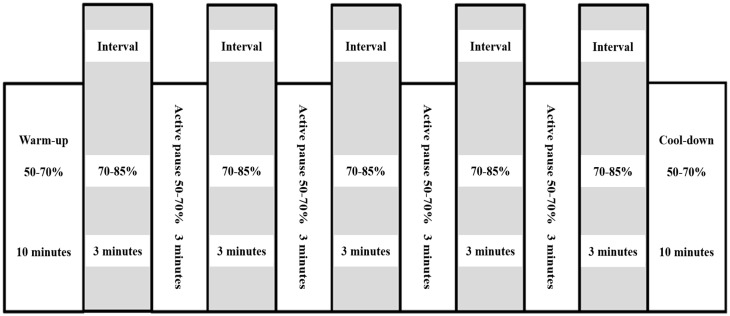
Schematic illustration of interval training for pulmonary rehabilitation.

The aerobic exercise program was conducted through interval training: a 10-min warm-up at 50–70% of the heart rate reserve (HRR), followed by five 3-min intervals of walking on a treadmill at 70–85% of the HRR, four 3-min walks at 50–70% of the HRR, and a 10-min cool down at 50–70% of the HRR. HRR is the difference between the resting heart rate (HR) and maximum HR and was calculated using the results of each subject’s first cardiopulmonary exercise test (CPET) data. A schematic illustration of interval training is provided in Figure 1. Adjustments were made so that all patients maintained the target HR at equivalent HRR percentages. Metabolic equivalents were calculated from the speed and slope of the treadmill, which were adjusted continuously to ensure that every training session was performed at the assigned HR. All training sessions were monitored by medical staff through electrocardiography, oxygen saturation and HR measurements, and measurement of the subjective rated perceived exertion (RPE) and modified Medical Research Council score to reduce the risk of possible complications during exercise. To verify the safety of PR, a record of any adverse events, including major cardiovascular or other musculoskeletal complications, was noted by the medical staff whenever the aerobic exercise program was performed.

For resistance exercise, upper and lower extremity strengthening training was taught and implemented for 10 min following a rest period after the end of the aerobic exercise. Participants were told to repeatedly perform the resistance exercises at home.

### 2.3. Primary and Secondary Outcomes

The primary outcomes were differences in maximal oxygen uptake (VO_2_max) and six-minute walking distance (6MWD) during the six-minute walk test (6MWT) before and after the eight-week PR program. The secondary outcomes were differences in hemodynamic responses, pulmonary function test results, peak cough flow (PCF), St. George’s Respiratory Questionnaire (SGRQ) scores, peripheral muscle strength, and skeletal muscle mass (SMM) during the same period.

### 2.4. Exercise Capacity

#### 2.4.1. Cardiopulmonary Exercise Test

All study participants completed the CPET using a modified Bruce treadmill protocol. The CPET was conducted under the supervision of medical staff that was blinded to group assignment and using a real-time recording with a 12-channel electrocardiogram (CASE; GE Healthcare, Waukesha, WI, USA), respiratory gas analyzer (Quark-CPET, COSMED), automatic blood pressure (BP) and pulse monitor (Tango M2; SunTech Medical, Morrisville, NC, USA), and treadmill (T2100-ST2, GE Healthcare). VO_2_max, minute ventilation/carbon dioxide production (VE/VCO_2_) slope values, and oxygen saturation during exercise were measured using the respiratory gas analyzer. The maximal HR, resting HR, and maximal and resting systolic/diastolic BP were measured using the electrocardiogram. The rate pressure product (RPP) = (systolic blood pressure × heart rate), HRR, total exercise time, and Borg’s scale were also measured [11,12].

VO_2_max was defined as the highest 20-sec interval average measured during the last 1 min of the CPET. Early termination of CPET was determined using the American College of Sports Medicine guidelines: achievement of an RPE of 17 (hard to very hard) and a respiratory exchange ratio of >1.10. The follow-up CPET was performed in the same manner. 

#### 2.4.2. Six-Minute Walk Test

The 6MWT was performed in accordance with the ATS/ERS guidelines [13]. When the test was conducted, peripheral oxygen saturation (SpO_2_) at the start and end of the 6 MWT and the difference between the two values were calculated.

### 2.5. Pulmonary Function Test

In accordance with the ATS/ERS guidelines, spirometry was performed with a VMAX 22 spirometer (Sensormedics) in the sitting position. Forced vital capacity (FVC), forced expiratory volume in one second (FEV1), and diffusion capacity of the lung for carbon monoxide (DL_CO_) were evaluated [14]. 

PCF was assessed using an Asthma Mentor Peak flow meter (Respironics) [15]. The participants performed a rapid, explosive exhalation after a maximal inhalation in a sitting position. At least three tests were performed, and the maximum value was selected after the test.

### 2.6. Health-Related Quality of Life Assessment

We evaluated health-related quality of life using SGRQ [16]: a frequently used test that has been validated in patients with IPF [17]. The total score of the SGRQ and scores in each domain area (symptoms, activity, and impact) were calculated. The total score on the SGRQ is expressed on a scale of 0–100, with 0 representing the best health-related quality of life.

### 2.7. Muscle Strength Test and Bio-Electrical Impedance Analysis

Handgrip strength was measured using a JAMAR Plus+ hand dynamometer (Performance Health, Warrenville, IL, USA) [18]. The average value was calculated from three measurements on each hand. Isokinetic knee flexion and extension were tested at a velocity of 60 °/s and 120 °/s using an isokinetic dynamometer (BIODEX System 4 ProTM; BIODEX, Natcon Drive Shirley, NY, USA) [19]. A bioelectrical impedance analyzer (InBody S10; Biospace, Seoul, Korea) was used to measure SMM and phase angle (PhA) [20].

### 2.8. Statistical Analysis

Data analysis was performed using SPSS version 25.0 (SPSS Inc., Chicago, IL, USA). For baseline characteristics, continuous variables are expressed as either mean ± standard deviation or median (interquartile range) and an independent *t*-test or Mann-Whitney’s U test was used. Categorical variables are expressed as *n* (%), and the chi-square test or Fisher’s exact test was used. Independent *t*-testing or Mann-Whitney U test was used for between-group comparisons and paired *t*-test or Wilcoxon’s signed-rank test was used for intra-group comparisons. The significance level was set at *p* < 0.05.

## 3. Results

### 3.1. Patient Characteristics

**Figure 2:**
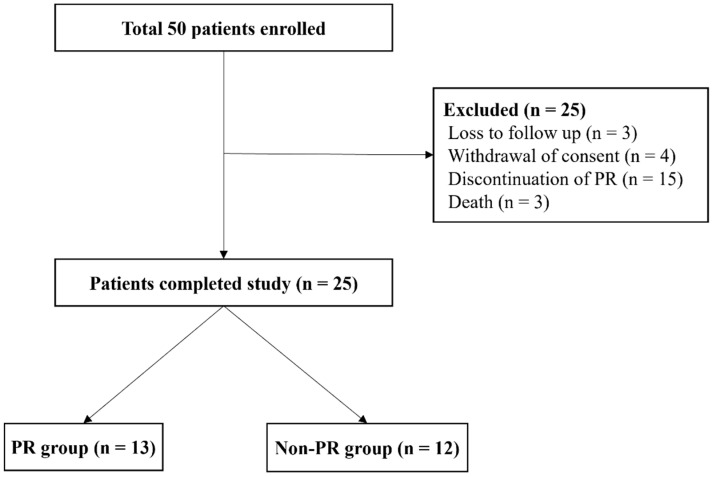
Flow chart of enrollment and analyzed population.

Originally, 50 patients who met the criteria were enrolled in this study, but 25 patients met the exclusion criteria or were lost to follow-up. Therefore, 25 patients completed the study, 13 patients in the PR group and 12 patients in the non-PR group (Figure 2).

#### PR, Pulmonary Rehabilitation

The mean age of the patients was 68 years old, and most subjects were male. Most of the patients had a history of smoking. The two groups did not differ significantly in any of the baseline clinical characteristics (Table 1). Most patients showed mild restrictive lung function defects on spirometry. Although the difference was not statistically significant, the PR group showed more severely restrictive lung function defects than the non-PR group.

### 3.2. Cardiopulmonary Exercise Test

In the PR group, the VO_2_max and VE/VCO_2_ slopes showed a significant improvement after eight weeks (*p* = 0.006 and *p* = 0.02, respectively), but the difference in the rate of change from that in the non-PR group was not statistically significant (*p* = 0.115 and *p* = 0.088, respectively) (Table 2). 

Elevated cardiovascular stress at CPET stage 3, as measured by RPP, was also significantly decreased in the PR group (−20.3% (−26.5–−15.1)), but again, the rate of change between the groups did not differ significantly (*p* = 0.051). The difference between the groups in the degree of change in HRR at three minutes was significant after eight weeks. (Inter-group *p* = 0.015). 

**Figure 3:**
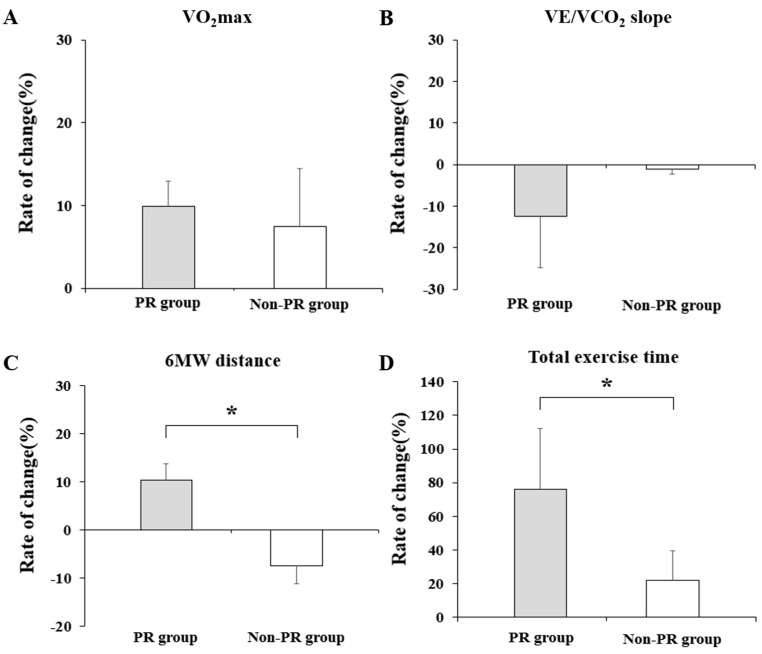
Key results of the cardiopulmonary exercise test, six-minute walk test in the PR and the non-PR groups after eight weeks (**A**); Comparison of changes in VO_2_max between the PR group and the non-PR group (**B**); Comparison of changes in VE/VCO_2_ slope between the PR group and the non-PR group (**C**); Comparison of changes in 6MWD between the PR group and the non-PR group (**D**) Comparison of changes in total exercise time between the PR group and the non-PR group. Asterisk (*) indicates significance at *p* < 0.05. PR, pulmonary rehabilitation; VO_2_max, maximal oxygen uptake; VE/VCO_2_, minute ventilation/carbon dioxide production; 6MWD, six-minute walking distance.

The change in total exercise time after eight weeks of PR was 76.2% (24.2–59.1) in the PR group and 21.9% (−7.–22.5) in the non-PR group, showing a statistically significant increase in the PR group (*p* = 0.005) (Figure 3).

### 3.3. Six-Minute Walk Test

After eight weeks of PR, 6MWD improved significantly in the PR group, and the difference between the groups was also statistically significant (Inter-group *p* = 0.002). In the case of the lowest SpO2, the median value rather decreased after eight weeks in the PR group, but the rate of change itself differed significantly from that of the non-PR group (Inter-group *p* = 0.017) (Table 3).

### 3.4. Pulmonary Function Test

In the intra-group comparison before and after the eight-week PR program, FVC, FEV1, FEV1/FVC, and DLco did not change significantly in either group (Table 4). However, PCF changed significantly in the PR group (*p* = 0.001). 

### 3.5. Saint George’s Respiratory Questionnaire Scores

The SGRQ score did not change significantly in any domain (or the total score) in either the PR after the eight-week PR program or the non-PR group, and the between-group comparison showed no significant difference in the rate of change (Table 5). Six patients in the PR group and four patients in the non-PR group achieved the minimal clinically important difference, defined as a four-unit reduction in the total score [21].

### 3.6. Muscle Strength Test and Bio-Electrical Impedance Analysis

Grip power increased in the PR group after eight weeks and decreased in the non-PR group (Table 6). For the left hand, there was a statistically significant difference between the groups (16.1 (4.9–32.1) vs. −2.8 (−7.5–7.9), *p* = 0.007). Total SMM increased in both groups after eight weeks, but the change was not statistically significant. The PhA of the left lower extremity differed significantly between the groups and the difference between the two groups was significant (0.2% (−4.3–4.9) vs. −7.5% (−10.4–−1.8), *p* = 0.022).

### 3.7. Safety Profile

The eight-week PR program was conducted under the supervision of one or more rehabilitation medicine physicians and nursing specialists using monitoring devices. No adverse events occurred during the PR program.

## 4. Discussion

This study evaluated the effectiveness of PR in patients with IPF. Our results demonstrate improvements in exercise capacity, as shown by the 6MWD, VO_2_max, and VE/VCO_2_ slopes, after eight weeks of PR. However, the PFT, clinical symptoms, and quality of life measured by the SGRQ did not change. No adverse events, including major cardiovascular/musculoskeletal or other severe complications, were observed in the PR group.

The efficacy of PR in COPD has been proven in many clinical studies, and rehabilitation programs have been widely adopted to treat patients with COPD [22]. Although the effectiveness of PR in non-COPD patients has been reported, studies on restrictive pulmonary diseases such as IPF are lacking [23]. Symptoms such as dyspnea, which patients complain of subjectively, are difficult to improve and remain a major challenge in clinical practice. Therefore, we conducted this study to investigate the efficacy of PR in patients with IPF.

This study demonstrated the efficacy of PR in patients with IPF, who showed significant improvements in VO_2_max during the CPET. The direct measurement of VO_2_max is the best indicator of aerobic health, and changes in aerobic health are associated with changes in mortality [24]. In addition, because exercise capacity, measured as VO_2_max, is also known to be a strong factor related to survival time in healthy adults, a change in the mortality in the PR group can be expected [25]. Patients who completed our PR program also demonstrated improvements in 6MWD, PCF, and peripheral muscle strength. 

Previous randomized controlled studies using exercise training programs in patients with IPF and other forms of ILD showed improvements only in the 6MWT, which is a test performed at submaximal intensity [26,27]. In our study, patients were tested using the CPET and 6MWT, and those who participated in supervised PR improved both their submaximal (6MWT) and maximal exertion (VO_2_max) levels. Our findings are consistent with those of Holland et al. [26] but extend further with additional outcomes they did not observe, such as improved VO_2_max, PCF, and peripheral muscle strength. Nishiyama et al. reported similar results in a small series of patients with IPF who underwent a nine-week PR program [27]. That study had several limitations, including the small number of patients and the lack of blinding of the investigators. More recently, Vainshelboim et al. showed that a 12-week training program for patients with IPF could result in a meaningful improvement in the 6MWT, cardiorespiratory fitness expressed as VO_2_max, and aerobic endurance (anaerobic threshold) measured objectively using the CPET [28]. Among ILDs, IPF together with asbestosis could represent diseases with the potential for clinically meaningful benefits from PR, as recently reported in an elegant article by Dowman et al. [29]. 

Functional exercise capacity is an important measure for evaluating and monitoring patients with cardiopulmonary disease. The 6MWT has been broadly used in clinical settings because of its patient tolerability, ease of implementation without the need for special equipment, and good correlation with patient outcomes [30]. In our study, we observed that exercise capacity, measured by the 6MWD, showed significant improvement in the PR group directly following PR. To our knowledge, previous PR studies have generally included patients with ILD, only a subgroup of whom had IPF. Because it is known that the magnitude of improvements in the 6MWD is less pronounced in IPF than in other ILD etiologies, it is difficult to compare our results with those of previous studies [26]. In this study, the PR group included only IPF patients, and they showed significant improvement. Previous studies reported similar results: outpatient PR and a standalone exercise training programs with a duration longer than eight weeks produced improvements in 6MWD of 25 m and 35 m, respectively [26,29]; however, the magnitude of those improvements was weaker than in our study. A Cochrane analysis found a mean 6MWD difference of +44 m in ILD and +36 m in IPF patients after PR [31]. 6MWD and a change in patients with IPF have been used as independent predictors of mortality [32]. Therefore, the improvement of 6WMD in this study might suggest not only an improvement in exercise capacity but also a survival benefit. A follow-up study that can confirm a survival benefit from PR through long-term follow-up is warranted. 

We did not confirm an improvement in symptoms or a change in SGRQ scores after eight weeks of PR, which differs from a previous study that showed improvement in the total SGRQ and three domain (symptoms, activity, and impact) scores after 12 weeks of PR in COPD patients [33]. We attribute that discrepancy to our small number of subjects, non-randomized controlled study (RCT) design, and relatively short PR period. In addition, the characteristics of chronically progressive IPF might have had an effect.

The actual mechanism for PR in patients with IPF is not well understood. Using a mouse model, Wang et al. reported that regular aerobic exercise for eight weeks improved high-fat diet-associated pulmonary fibrosis, as shown by several profibrogenic factors, including transforming growth factor-β (65.5 pg/mL vs. 41.3 pg/mL, *p* = 0.013) in the bronchoalveolar fluid after exercise [34]. Those authors suggested that regular aerobic exercise might improve pulmonary fibrosis in mice models by counteracting insulin resistance, chronic inflammatory response, and pro-oxidative/anti-oxidative imbalance.

This study has several limitations. First, it was a single-center, prospective interventional study with a small number of patients. However, the baseline characteristics of the patients were similar to those in previous studies, and the PR group and the non-PR groups did not differ significantly. Second, we did not evaluate the long-term effects of the PR program in patients with IPF. Because our PR program was conducted three times a week on an outpatient basis, it was difficult to sustain it for a long time. Further studies are needed to identify the long-term effects of PR programs on IPF patients. Third, due to selection bias, all of our subjects had IPF of mild severity, characterized by preserved FVC and SpO_2_ at rest without significant hypoxemia during 6MWT, so our result might be difficult to generalize to all patients with IPF. A large-scale RCT to demonstrate the effectiveness of PR in patients with IPF is needed.

## 5. Conclusions

In this study, PR for eight weeks in patients with IPF showed significant improvements in the VO_2_max and 6MWD of patients with IPF compared with the non-PR group. However, PFT, clinical symptoms, or impact on quality of life measured by the SGRQ did not differ between the groups. No adverse events, including major cardiovascular/musculoskeletal or other severe complications, were identified in the PR group.

## Figures and Tables

**Table 1:** Baseline characteristics of study subjects.

	Total (*n* = 25)	PR Group (*n* = 13)	Non-PR Group (*n* = 12)	*p*-Value
Male	23(92%)	11(85%)	12(100%)	0.480
Age (year)	68 ± 5.5	68 ± 5.3	69 ± 5.9	0.430
Ever smoker	23(92%)	11(85%)	12(100%)	0.480
BMI (kg/m^2^)	25 ± 3.4	25 ± 4.4	25 ± 2.2	0.915
FVC, % predicted	78.3 (69.5–88.5)	73.7 (63.5–80.5)	83.2 (76.2–91.5)	0.062
FEV1, % predicted	85.1 (74.5–94.5)	82.6 (72.0–89.5)	87.8 (76.5–98.5)	0.361
FEV1/FVC, %	77.1 (71.5–84.0)	79.9 (77.0–85.0)	74.0 (70.2–79.7)	0.059
DLco, % predicted	63.2 (57.5–70.0)	64.3 (56.5–71.5)	62.0 (57.2–68.7)	0.642
VO_2_max, mL·kg^−1^·min^−1^	21.3 (18.9–24.5)	22.6 (20.1–24.9)	19.8 (15.4–23.3)	0.071
LVEF (%)	64.0 (61.2–68.0)	66.2 (63.0–69.0)	62.8 (58.5–67.0)	0.259
mMRC	1.0 (1.0–2.0)	1.1 (1.0–2.0)	1.0 (0.2–1.7)	0.591
SGRQ_Total	27.0 (16.1–33.5)	23.9 (16.4–30.7)	30.3 (15.9–40.6)	0.339
Anti-fibrotic drug use	25(100%)	13(100%)	12(100%)	-
Hazardous Substance Exposure	3(12%)	3(23%)	0(%)	0.220
Family history	0(0.0%)	0(0.0%)	0(0.0%)	-

Values are presented as mean ± standard deviation and median (interquartile range) or number (%). PR, pulmonary rehabilitation; BMI, body mass index; FVC, forced vital capacity; FEV1, forced expiratory volume in 1 s; DLco, diffusing capacity of the lungs for carbon monoxide; VO_2_max, maximal oxygen uptake; LVEF, left ventricular ejection fraction; COPD, chronic obstructive pulmonary disease; CVA, cerebrovascular accident; CVD, cardiovascular disease; mMRC, modified Medical Research Council; SGRQ, St. George’s Respiratory Questionnaire.

**Table 2:** Results of cardiopulmonary exercise test before and after PR.

Variables	PR Group (*n* = 13)				Non-PR Group (*n* = 12)				Inter-Group *p*-Value		
	Baseline	8 Weeks Later	Rate of Change (%)	*p*-Value	Baseline	8 Weeks Later	Rate of Change (%)	*p*-Value	Baseline	8 Weeks Later	Rate of Change (%)
VO_2_max mL·kg^−1^·min^−1^	23 (20–25)	25 (23–28)	10 (2–19)	0.006	20 (15–23)	21 (16–25)	7 (−3–8)	0.508	0.071	0.041	0.115
VE/VCO_2_ slope	37 (32–39)	32 (27–37)	−12 (−26–1)	0.020	40 (32–49)	39 (34–42)	−1 (−9–9)	0.679	0.271	0.054	0.088
RPP stage 3	21491 (18,868–23,998)	17240 (14,457–20,039)	−20 (−26–−15)	0.003	19,619 (17,718–20,925)	17,448 (13,342–19,892)	−4 (−18–3)	0.459	0.341	0.916	0.051
HRR at 1 min	8 (4–10)	10 (5–14)	60 (−11–133)	0.460	13 (5–14)	14 (5–16)	51 (−52–49)	0.720	0.340	0.827	0.149
HRR at 3 min	22 (11–31)	34 (25–45)	79 (−4–124)	0.015	26 (4–39)	29 (19–38)	0 (−17–17)	0.391	0.596	0.459	0.015
Total exercise time	557 (471–642)	809 (740–844)	76 (24–59)	0.001	576 (316–754)	637 (445–778)	22 (−7–22)	0.346	0.821	0.017	0.005
HR rest	81 (65–94)	79 (66–87)	−1 (−5–9)	0.454	78 (68–79)	79 (67–84)	2 (−8–12)	0.642	0.430	0.890	0.509
HR max	139 (127–158)	144 (132–160)	4 (0–10)	0.042	131 (106–150)	135 (126–152)	4 (−3–7)	0.270	0.461	0.386	0.974
RER at VO_2_max	0.9 (0.9–1)	1 (1–1.1)	11 (3–15)	0.003	0.9 (0.8–1)	0.9 (0.8–1)	2 (−10–21)	0.640	0.230	0.056	0.193

Values are presented as median (interquartile range). PR, pulmonary rehabilitation; VO_2_max, maximal oxygen uptake; VE/VCO_2_, minute ventilation/carbon dioxide production; RPP, rate pressure product; HRR, heart rate recovery; HR, heart rate; RER, respiratory exchange ratio.

**Table 3:** Results of six-minute walk test before and after PR.

Variables	PR Group (*n* = 13)				Non-PR Group (*n* = 12)				Inter-Group *p*-Value		
	Baseline	8 Weeks Later	Rate of Change (%)	*p*-Value	Baseline	8 Weeks Later	Rate of Change (%)	*p*-Value	Baseline	8 Weeks Later	Rate of Change (%)
6MWD	491 (441–541)	537 (499–568)	10 (−1–21)	0.013	532 (505–553)	492 (447–544)	−7 (−12–1)	0.075	0.057	0.067	0.002
Initial SpO_2_	96 (94–98)	95 (94–96)	−1 (−2–1)	0.086	97 (96–98)	97 (96–99)	0 (0–1)	0.666	0.142	0.029	0.237
Lowest SpO_2_	91 (88–95)	89 (85–94)	−3 (−5–0)	0.027	92 (92–96)	93 (92–96)	0 (−1–2)	0.435	0.269	0.080	0.017
SpO_2_ difference	4 (3–6)	6 (3–9)	43 (−12–75)	0.035	5 (1–5)	4 (2–4)	−6 (−50–0)	0.437	0.227	0.114	0.051

Values are presented as median (interquartile range). PR, pulmonary rehabilitation; 6MWD, six-minute walking distance; SpO_2_, peripheral oxygen saturation.

**Table 4:** Results of pulmonary function test before and after PR.

Variables	PR Group (*n* = 13)				Non-PR Group (*n* = 12)				Inter-Group *p*-Value		
	Baseline	8 Weeks Later	Rate of Change (%)	*p*-Value	Baseline	8 Weeks Later	Rate of Change (%)	*p*-Value	Baseline	8 Weeks Later	Rate of Change (%)
FVC, % predicted	73.7 (63.5–80.5)	72.8 (63.5–81.5)	−0.3 (−8.5–5.8)	0.651	83.2 (76.2–91.5)	83.9 (73.7–93.7)	0.9 (−3.5–5.1)	0.643	0.062	0.018	0.717
FEV1, % predicted	82.6 (72.0–89.5)	80.9 (74.0–86.0)	−1.6 (−7.2–4.1)	0.367	87.8 (76.5–98.5)	88.6 (79.2–97.5)	1.0 (−5.0–6.6)	0.622	0.361	0.170	0.401
FEV1/FVC	79.9 (77.0–85.0)	78.3 (76.0–83.0)	−1.8 (−4.1–0.6)	0.062	74.0 (70.2–79.7)	73.5 (68.5–78.7)	−0.6 (−3.5–2.2)	0.430	0.059	0.085	0.358
DLco, % predicted	64.3 (56.5–71.5)	61.8 (54.5–72.0)	−3.5 (−12.0–1.3)	0.135	62.0 (57.2–68.7)	61.9 (51.2–73.2)	0.7 (−11.2–9.7)	0.940	0.642	0.988	0.183
PCF	323.0 (250.0–395.0)	426.1 (350.0–475.0)	35.5 (16.6–57.1)	0.001	414.1 (325.0–485.0)	456.6 (367.5–510.0)	18.7 (−4.2–16.1)	0.208	0.039	0.427	0.006
mMRC	1.1 (1.0–2.0)	0.9 (1.0–1.0)	−13.6 (−50.0–0.0)	0.083	1.0 (0.2–1.7)	1.0 (0.2–1.7)	0.0 (0.0–0.0)	-	0.591	0.774	0.098

Values are presented as median (interquartile range). PR, pulmonary rehabilitation; FVC, forced vital capacity; FEV1, forced expiratory volume in one second; DLco, diffusion capacity of the lung for carbon monoxide; PCF, peak cough flow; mMRC, modified Medical Research Council.

**Table 5:** Results of SGRQ before and after PR.

Variables	PR Group (*n* = 13)				Non-PR Group (*n* = 12)				Inter-Group *p*-Value		
	Baseline	8 Weeks Later	Rate of Change (%)	*p*-Value	Baseline	8 Weeks Later	Rate of Change (%)	*p*-Value	Baseline	8 Weeks Later	Rate of Change (%)
SGRQ_Symptom	32.7 (18.5–49.5)	32.3 (11.9–51.3)	10.0 (−23.5–33.7)	0.861	34.5 (15.1–44.8)	31.4 (21.5–41.3)	3.4 (−46.5–55.7)	0.466	1.000	0.910	0.786
SGRQ_Activity	39.6 (29.6–55.5)	34.7 (17.8–50.6)	−21.5 (−37.6–0.0)	0.250	46.0 (29.8–66.1)	47.4 (31.2–65.4)	3.0 (−16.6–20.7)	0.660	0.500	0.167	0.083
SGRQ_Impact	12.2 (6.4–17.8)	12.0 (2.9–19.7)	5.7 (−67.4–63.5)	0.924	20.0 (5.0–27.9)	18.5 (4.9–26.6)	37.1 (−43.5–64.9)	0.790	0.683	0.237	0.862
SGRQ_Total	23.9 (16.4–30.7)	22.3 (11.5–33.4)	−4.6 (−26.8–12.0)	0.274	30.3 (15.9–40.6)	29.5 (14.8–38.4)	8.1 (−28.1–43.1)	0.796	0.339	0.239	0.386

Values are presented as median (interquartile range). PR, pulmonary rehabilitation; SGRQ, St. George’s Respiratory Questionnaire.

**Table 6:** Results of peripheral muscle strength and SMM before and after PR.

Variables	PR Group (*n* = 13)				Non-PR Group (*n* = 12)				Inter-Group *p*-Value		
	Baseline	8 Weeks Later	Rate of Change (%)	*p*-Value	Baseline	8 Weeks Later	Rate of Change (%)	*p*-Value	Baseline	8 Weeks Later	Rate of Change (%)
Grip power (Right)	30.3 (26.5–35.9)	33.1 (28.05–40.10)	8.4 (0.72–20.45)	0.144	35.1 (31.25–41.20)	34.1 (31.0–41.6)	−2.9 (−13.1–5.1)	0.406	0.112	0.739	0.065
Grip power (Left)	28.3 (23.8–33.8)	32.0 (27.25–36.65)	16.1 (4.92–32.10)	0.005	33.6 (24.13–41.00)	31.8 (24.6–38.5)	−2.8 (−12.0–8.7)	0.335	0.096	0.928	0.007
SMM_Total	26.5 (23.1–30.1)	26.6 (22.80–30.40)	0.4 (−1.25–2.14)	0.670	28.4 (24.93–31.98)	28.7 (25.0–31.9)	2.2 (−2.9–3.3)	0.959	0.320	0.216	0.513
SMM_Right UEx	2.6 (2.2–3.1)	2.6 (2.23–3.01)	−2.1 (−4.41–0.74)	0.078	2.9 (2.62–3.46)	3.0 (2.5–3.5)	6.0 (−2.8–10.4)	0.656	0.197	0.025	0.242
SMM_Left UEx	2.6 (2.2–3.0)	2.5 (2.19–2.95)	−2.1 (−3.92–0.18)	0.028	2.9 (2.56–3.25)	3.0 (2.5–3.4)	4.8 (−3.8–8.7)	0.534	0.221	0.097	0.341
SMM_Right LEx	7.5 (6.4–8.4)	7.6 (6.45–8.80)	2.2 (−0.32–4.43)	0.023	7.6 (6.8–8.6)	8.1 (7.1–9.3)	7.8 (−1.0–6.2)	0.213	0.769	0.404	1.000
SMM Left LEx	7.4 (6.5–8.5)	7.6 (6.49–8.94)	2.5 (0.93–4.74)	0.009	7.6 (6.7–8.5)	8.1 (7.2–9.1)	8.2 (−2.1–6.0)	0.286	0.735	0.374	0.828
PhA Right UEx	5.6 (5.1–6.0)	5.5 (5.10–6.00)	−0.3 (−3.44–2.05)	0.660	5.8 (5.5–6.3)	5.6 (5.2–6.0)	−3.3 (−6.5–1.3)	0.073	0.319	0.796	0.216
PhA Left UEx	5.4 (5.1–5.7)	5.3 (4.95–6.00)	−1.1 (−4.81–1.80)	0.387	5.6 (5.2–6.2)	5.4 (4.9–6.0)	−3.1 (−8.3–2.4)	0.076	0.535	0.854	0.397
PhA Right LEx	5.9 (5.0–6.9)	5.9 (5.00–6.80)	0.0 (−6.95–5.49)	0.787	5.8 (5.0–6.5)	5.4 (4.7–6.1)	−6.3 (−9.4–0.0)	0.024	0.751	0.187	0.056
PhA Left LEx	5.9 (5.1–6.9)	5.8 (5.15–6.70)	0.2 (−4.35–4.90)	0.865	5.7 (5.0–6.4)	5.2 (4.6–6.1)	−7.5 (−10.6–−1.7)	0.006	0.672	0.076	0.022

Values are presented as median (interquartile range). SMM, skeletal muscle mass; PR, pulmonary rehabilitation; UEx, upper extremity; LEx, lower extremity; PhA, phase angle.

## Data Availability

All the data are contained within the manuscript.
